# A university’s response to people with disabilities in Worcester, Western Cape

**DOI:** 10.4102/ajod.v8i0.439

**Published:** 2019-10-24

**Authors:** Jana V. Müller, Lieketseng Ned, Hananja Boshoff

**Affiliations:** 1 Ukwanda Centre for Rural Health, Rural Clinical School, Faculty of Medicine and Health Sciences, Stellenbosch University, Cape Town, South Africa; 2 Centre for Rehabilitation Studies, Faculty of Medicine and Health Sciences, Stellenbosch University, Cape Town, South Africa

**Keywords:** clinical training, collaboration, community engagement, disability, distributed training, undergraduate health sciences

## Abstract

**Background:**

The call for institutions of higher education to foster interaction with communities and ensure training is responsive to the needs of communities is well documented. In 2011, Stellenbosch University collaborated with the Worcester community to identify the needs of people with disabilities within the community. How the university was engaging with these identified needs through student training still needed to be determined.

**Objectives:**

This study describes the engagement process of reciprocity and responsivity in aligning needs identified by persons with disability to four undergraduate allied health student training programmes in Worcester, Western Cape.

**Method:**

A single case study using the participatory action research appraisal methods explored how undergraduate student service learning was responding to 21 needs previously identified in 2011 alongside persons with disability allowing for comprehensive feedback and a collaborative and coordinated response.

**Results:**

Students’ service learning activities addressed 14 of the 21 needs. Further collaborative dialogue resulted in re-grouping the needs into six themes accompanied by a planned collaborative response by both community and student learning to address all 21 needs previously identified.

**Conclusion:**

Undergraduate students’ service learning in communities has the potential to meet community identified needs especially when participatory action research strategies are implemented. Reciprocity exists when university and community co-engage to construct, reflect and adjust responsive service learning. This has the potential to create a collaborative environment and process in which trust, accountability, inclusion and communication is possible between the university and the community.

## Introduction

Rural health science training is not a new concept, particularly for medical students globally. The earliest account of this in South Africa was in KwaZulu-Natal in the 1940s and later in 1992 with the Allied Health Sciences (AHS). In a study by Pillay et al. (2016) on AHS learners’ perceptions of rural clinical training in their final year of study, it was evident that rural training changes students’ experience practice from urban-based, individualised care to the more rural-based clinical training. Students found that rural clinical training is undoubtedly challenging for both the educators and the learners given its complexity. However, the necessity of undergraduate health science students to go through varied contextual training that is socially responsive cannot be overstated. The experiential learning offered by rural training not only aims to equip students with the necessary skill and insight to work within a community, but also enable them to identify and respond to needs at the community level. Of equal importance, this responsiveness in clinical training should facilitate some level of accountability in higher education institutions (HEIs) towards the communities they work with in training their students (Doherty & Couper 2016; Global Consensus for Social Accountability of Medical Schools 2015). This is particularly critical if we are to have a responsive curriculum within the context of disability and rehabilitation where vast capacity is needed to address diverse disability issues effectively.

Stellenbosch University’s (SU) Ukwanda Centre for Rural Health established the first rural clinical school (RCS) as an official rural campus to Tygerberg Faculty of Medicine and Health Sciences (FMHS) in 2011. The purpose of this school was to equip students with the skills and willingness to work in rural and underserved areas (Van Schalkwyk et al. 2011). Initially, only medical students were trained at the RCS, but in 2013 the school offered rotating rural programmes for all four health care training programmes, namely Occupational Therapy (OT), Physiotherapy (PT), Human Nutrition (HN) and Speech Language and Hearing Therapy (SLHT) (Pillay et al. 2016). This article focuses on these four divisions. Each of these divisions had their own outcomes, divisional priorities and separate coordinators who were employed independently of each other and who had no overlap in academic responsibilities.

While the university was in the preparatory stages for the placement of students in this rural school, a conference was collaboratively hosted by the Faculty of Theology, the Centre for Rehabilitation Studies (CRS) and the Department of Psychology at SU with the theme Disability, Theology and Human Dignity. This was SU’s first international interfaculty conference. The third day of this conference was held at the campus of the National Institute for the Deaf in Worcester with the goal of meeting with a group of persons with disabilities in Worcester. The rationale for this was to get an understanding of the needs of persons with disability in Worcester as the FMHS at the time was busy developing an RCS for students’ rural placement. From this third day of the conference, a two-page list of needs was then developed and handed over to clinical facilitators of the above-mentioned divisions. These expressed needs are tabulated in Table 2 of this article. The first round of students’ placements was scheduled for 2012.

This was also a helpful internal process for SU to further discuss the issues of disability and human dignity within a broader university strategy. The Hope project, which is SU’s institutional response to issues of poverty and vulnerability in Africa, chimes closely to some of the 2011 conference recommendations and the needs that had been specified by persons with disabilities in Worcester. Subsequent to this, the CRS started planning how to ensure that clinical facilitators that are placing students in Worcester with the goal of responding to the needs of persons with disabilities in that area are using the list to guide them in placement of students (Ned et al. 2015).

This article therefore focuses on how academic coordinators responded to the list of needs as expressed by persons with disability through the students’ rural clinical practice blocks in Worcester.

### Background of the rural clinical school

The RCS is a small campus consisting of six clinical educators and ten support staff with an average of 50 students on the campus at any one time. Ukwanda Centre for Rural Health focuses on rural placement training, where the Worcester community, students and academic coordinators work closely together. The school encourages collaboration and innovative academic development. Students from the four disciplines are placed in their final year for short clinical block rotations. A portion of students are placed at rural sites and others at urban sites in a 1-year cycle (Pillay et al. 2016). Pillay et al. (2016) further reported that, on average, 44 of 100 SU AHS students experienced the RCS training platform during their final year of training. By 2014, RCS students were trained at 19 different sites in Worcester.

These sites included areas where persons with disabilities sought health care and sheltered work opportunities. These were centres specifically for persons with disability and centres for early childhood development. The sites for training were identified by the academic coordinators who were resident in the community and had been working with the local health system for between 2 and 10 years. The selection was based on needs analyses conducted with the staff at potential training sites, prior to this current project and before the introduction of the CRS. Of the 19 sites, ten of them accommodated more than one division’s students at a time, allowing for Inter-professional education and collaborative practice (IPE) to occur at these sites. The 19 placement sites were predominantly primary health care and community-based services. The students were involved in service learning at clinics, mobile clinics, special schools, non-governmental organisations (NGOs), rehabilitation centres, early childhood development crèches, private practices, hospitals and primary schools.

Each division had their own academic objectives for the student rotations. The primary focus areas were as follows:

- *Physiotherapy* focuses on health promotion and disease prevention by giving exercise and information classes, training carers regarding ergonomic rehabilitation and assessment and treatment of patients on home visits.
- *Human Nutrition* focuses on health promotion at schools, clinics, on farms and the local radio station. Disease prevention also took place by means of talks, pamphlets, recipes, audits of food service units, assessments of patients, lifestyle education and monitoring of health facilities. Breastfeeding consultations, growth monitoring and recipe development formed part of the programmes.
- *Speech, Language and Hearing Therapy* focuses on community-based interventions and health promotion. Screening, assessment of adults and children, and provision of swallowing, speech and language therapy. Sign Language and language enrichment with children, assisting patients with post lingual hearing loss, aural therapy and articulation.
- *Occupational Therapy* focuses on paediatrics, particularly targeting early childhood development, psychosocial block with a particular focus on mental health, service learning which is about provision of community-based services and programme development, and lastly a general hospital block which focuses on one-on-one rehabilitative treatment.

Table 1 presents a summary of the length and type of block rotations each AHS division offered at the RCS for a period of 4 years since the divisional programmes started.

**TABLE 1:** Summary of divisional rotations.

Divisions (all final year students)	Physiotherapy	Human nutrition	Speech, language and hearing therapy	Occupational therapy
Year started	2011	2012	2013	2013
Types of rotations a student can do at the RCS in Worcester	Community block Paediatric neurology Adult neurology	Ukwanda block Community nutrition Food service management Therapeutic nutrition	Community block	Physical rehabilitation Psychosocial interaction Community interaction Learning and development
Length of rotations (weeks)	6	6	8	6
Number of rotations per year	5	4	3	4
Number of students per rotation	2–3	2–4	8–10	2

RCS, rural clinical school.

The objective of this study was to describe the process the university (divisional clinical coordinators in collaboration with the CRS) followed to engage with the needs of persons with disabilities in the Worcester community, through the placement of the AHS students. There is less documentation of the engagement process that takes place between the university and the community. More specifically, this article, firstly, describes the university’s participatory process of responding and, secondly, identifies what is the list of needs that was responded to by the AHS student training at the RCS from 2011 to 2013, followed by recommendations by the community moving forward and plans implemented since.

## Methodology

A single participatory case study in Worcester with persons with disabilities and the community was undertaken following the list of needs they expressed in 2011. Framed within a participatory action research (PAR) approach, this case study methodology was particularly suited with the intention to gain an in-depth understanding into how the undergraduate rural student service learning was unfolding and responding to the expressed needs in this context (Stake 2008).

The study site has well-established disability institutions such as National Institute for the Deaf (NID), Institute for the Blind and DEAFNET with international footprints, exposing the various stakeholders that are also involved in continuously addressing needs of persons with disabilities. The list expressed by persons with disabilities highlighted a case for exploration of a collaborative and coordinated response to these expressed needs. It is within this context that the case was formulated. The rurality of the Worcester area within the Western Cape Province also added another context to the case formulation. This case study was the response of the academic coordinators to the expressed list of needs of persons with disabilities. The participatory nature and inclusion of various stakeholders acted as a better fit with the case study in shedding light on multiple perspectives relating to a collaborative response to the needs (O’Leary 2017).

In this regard, this tradition of qualitative inquiry was deemed appropriate to use while situating each stakeholder in its historical, political, economic and socio-cultural contexts demanding multiple data sources. This case study was approached in a manner suggested by Stake (2008) who viewed it mainly as the ‘object of study’ and not so much how the case is studied. Thus, PAR as an additional lens guided the collaborative process and approach of defining what the case was about from multiple perspectives and methods (Atkinson & Hammersley 1998; Le Compte & Schensul 1999). Participatory action research methods thus became the approach of the university’s response to the list of needs expressed by persons with disabilities (Ned et al. 2015). This is because PAR methods enable people to conduct their own analysis of the situation, plan and take action (Booi 2012). This study included a series of workshops using methods such as Venn diagrams for monitoring how the service learning is responding to the needs identified and the nominal group technique for prioritising needs for action planning.

Ontologically and epistemologically, this process was couched within the transformative paradigm which allows for recognition of unequal power held by theorists, the researcher and the participants who may hold dissonant perspectives on disability needs and how to effectively respond. This was an attempt to deconstruct the subjugated views (Henning &Van Rensburg 2004; Booi 2012) about addressing disability issues often perpetuated by dominant rhetoric. It was also an attempt to close the power gap in processes of knowledge production in order to strengthen the voices and actions of persons with disabilities who are often marginalised (Booi 2012; Chilisa 2012). The above-mentioned participatory methods like Venn diagrams and nominal group techniques in interactive workshops sessions were used to facilitate voices of persons with disabilities (Ned et al. 2015). Various studies have shown these participatory methodologies to be effective in engaging community participants through interactive workshop sessions especially in shifting power dynamics in research and to ensure that the marginalised speak about their experiences and speak back (Mitchell, De Lange & Moletsane 2017). Additionally, these participatory methods are known to privilege the meaningful participation of the community. The principal researcher from the CRS facilitated these interactive workshops. Following disabled people’s slogan of nothing about us without us, it was thus important that persons with disabilities are at the centre of discussions with the process allowing for a back and forth process of ongoing feedback to each group involved. These engagements happened in a series of interactive workshops (Ned et al. 2015). First, persons with disabilities were invited to validate the list of needs before it was given to the academic coordinators. Second, the AHS coordinators presented their response to the needs in an audience of persons with disabilities and their organisations. This presented another shared dialogue opportunity where persons with disabilities shared their reflections of the response from the academic coordinators. The principal researcher from the CRS acted as a facilitator in these workshops. This shared dialogue led to a further synthesis of the needs of persons with disabilities. Lastly, another workshop took place about plans for a collective way forward to ensure that these needs are addressed. Thus, the participatory nature of PAR allowed for sharing of knowledge between groups and was an important aspect of ethics (ensuring research that is beneficial to and inclusive of the community and respecting the autonomy of the community while also emphasising equal participation). This implies that reporting was not a once-off exercise left for the end of the research process but became a continuous process throughout these engagements as allowed by the PAR method of action reflection.

We applied for ethical clearance from the university and were informed that ethical clearance was not needed and advised to rather apply when interventions begin. The project was interpreted as consultative workshop which does not need ethical approval. Although participatory research is inherently ethically good compared to traditional research, it is also prone to issues of unequal power relations which may result in ethical challenges in partnerships. Thus, the feedback from ethics was concerning particularly because it may speak to poor recognition of PAR as a valid research methodology within the health sciences. This project had an advisory committee which consisted of different representatives from disabled people’s organisations in Worcester. This committee served (amongst other things) to coordinate the activities of the project as well as to ensure the participation and inclusion of persons with disabilities.

Further details are described in the following sections explaining the steps taken in this engagement process.

### The participatory response: Action and reflection

The RCS AHS coordinators were tasked with preparing a response to how the students’ clinical rotations had been developed around the original needs identified for people with disability. These coordinators received the two-page list of needs for the first time in 2014 and were asked to assess how the AHS students’ training at the RCS was addressing some of these needs identified in 2011. Despite seeing the document for the first time, since the AHS coordinators who were only involved at the RCS after 2011, they engaged with the list of 21 needs, and compared them to the objectives of the clinical activities and the placement of the students. Alignments were noted, gaps were identified and their reflective response was presented to the Worcester community on 27 June 2014. The audience included persons with disabilities from the community, various representatives of disabled people’s organisations and various representatives from service providers across sectors, local government and the academic coordinators. The scope of this work did not document the type of disabilities which were represented in the audience. However, disabled people’s organisations that were present mainly focused on visual, physical and hearing impairments.

### The reflections of the coordinators

Concerning the above-mentioned reflection of how the clinical programmes might be able to meet some of the identified needs expressed during the disability conference in 2011, it was revealed that the service learning of students at Ukwanda spoke to 14 of the 21 needs identified, a summary of which is presented in Table 2. This table presents needs identified together with activities of students and staff as a response.

**TABLE 2:** List of needs identified with students and staff activities.

List of needs identified in 2011	Influence of RCS students on meeting these needs	Influence of RCS staff on meeting these needs	Gaps identified and suggestions going forward to meet the needs identified
1. A work group be established to take forward issues and suggestions arising from presentations and discussions during the conference	No response	A working group consisting of both the CRS and RCS staff was formed to mediate issues between the faculty to guide the training of students and how students could engage with the community	Innovative solutions required for the different outcomes for each division, different planning times, different sites, different rotations The RCS staff to be more involved in issues that are integrated within the curricula as they engage with the students and the community of Worcester A working group developed with different stakeholders from the disability sector, government institutions and local government as well as the university needed to be established
2. A reference committee consisting of representatives of the seven disability groupings for the workgroup be selected	Community participation and mobilisation to align needs with training using community-based needs analysis	No response	The DoH raising issues that community service therapists lack basic skills for working in rural settings post their training
3. Clarify terminology	Understanding of disability and the related conceptual frameworks	Understanding of disability and the related conceptual frameworks	There was a need to plan the offering of accredited workshops to keep students up to date with the recent understandings and underpinnings of disability There was a need to design a course for the students on understanding disability and how to manage disability issues outside the medical model. The ICF endorsed by the WHO (2001) was used to facilitate holistic thinking and assessment of needs specific to the patient
4. Include in ECD and primary education the support of parents to enrich parenting skills in raising their children with disability	ECD screening at Empilisweni Clinic performed by all AHS, including education to mothers via private discussion and talks in the waiting area, Vukuhambe Centre for Children with Disability were assessed and treated by PT and SLHT students as well as at ASD where they worked with the PT students, PT students were also involved with children with disability and their parents at Breede Valley APD	HN, SLHT and PT coordinators included ECD in assessment criteria and outcomes for the students rotation in Worcester	OT students were unable to assist with ECD at Vukuhambe, ASD and APD due to other clinical commitments
5. Include aspects of disability in curricula of all levels of education	No response	SLHT coordinator was aware and involved in disability education in second and third year teaching	Important for the RCS coordinators to have a better idea of the theoretical training of the students prior to their arrival at the RCS for practical training as the HN, PT and OT coordinators were not aware how and when this is done during the undergraduate theoretical curriculum Also perhaps to have input in the effect of this training and possible ways for further development
6. Listing of needs identified by conference participants, e.g. mobility matters, access to entire building and not just part of it, transport, interpretation preferences	No response	Physical: Accessible Attitudinal: Social responsibility, acceptance and sensitising students, staff and community	Information: No facilities (Braille, Sign Language and Loop systems) Communications: Not fully accessible for people with sensory disabilities
7. Needs assessment of persons with disabilities in rural communities involving municipalities and government departments	Intervention based on needs of persons with disabilities assessed during home visits/consultations/Hospice Rehab centre and academic hospital ward round HN, PT, OT, SLHT and Medics – Use of the ICF to assess and refer patients	All coordinators had to do a needs analysis of the Worcester area including NGO, DoH input prior to student placement PT and OT students involved in a specific service learning project which is based on a thorough needs analysis All AHS coordinators involved in regular meetings with DoH regarding needs identified	More regular contact with DoE and DSD as well as municipality
8. Assessment of resources to appropriately address the identified needs, keeping in mind the uniqueness of the needs of different disabilities	At each of the sites mentioned below, the involved disciplines specifically assessed needs on a health, social and environmental level: HN, PT, OT, Medics, SLHT – Avian Park PT, SLHT – Hospice Rehab Centre HN – Nutrition quality monitoring at DoH clinics PT – Ergonomic assessments and individual home visits	Staff encouraged students to evaluate and refer patients using forms based on the ICF framework This helped students evaluate a holistic bio-psycho-social-spiritual plan management	Mobility, access and transport – Major issues for sustainability of projects PT recommend rural assistive devices, because roads can be inaccessible, no transport for persons with disabilities (e.g. dial-a-ride)
9. Sharing of resources between all stakeholders	No response	Human resources were shared: Avian Park Service Learning Centre, all sites where students are involved. Physical resources were shared: Avian Park Service Learning Centre was shared with community members (soup kitchen and swop shop) and Hospice RCS Campus: Multisectoral action team and DoH could make use of facilities for free if staff members were invited	The coordinators realised on reflection that each of the sites where their students are placed also share resources by means of access to files, rooms for treatment or admin, sometimes also staff
10. Research strategies to put theory into practice in ways that meet the needs of people with disabilities in rural areas	The following studies and presentations were given by students: OT – Functioning of stroke patients post-discharge from hospital Medics, PT, HN – Chronic obstructive pulmonary disorder, a study on the effect of an inter-professional management plan in Avian Park community PT – Management of chronic lower back pain in rural areas SLHT – Feeding of children with cerebral palsy	No research unit available at RCS	Opportunity – Shop front for research enquiries where community can approach the university via the rural clinical school to request research in a certain area of need Training of RCS staff to engage with research related to their environments
11. Equal study opportunities for people with disabilities	Centre for Student Counselling and Development (Disability Unit) on main campus in Stellenbosch RCS campus: Physical: accessible Attitudinal: Social responsibility, acceptance and sensitising students, staff and community	RCS campus: Physical: accessible Attitudinal: Social responsibility, acceptance and sensitising students, staff and community	RCS campus: Information: no facilities (Braille, Sign Language and Loop systems) Communications: not fully accessible for people with sensory disabilities
12. (a) Joint efforts in public education and training in communities regarding awareness and sensitisation	HN, OT, SLHT, PT – At all sites where students are clinically involved the community assists with their practical training PT, HN, OT, SLHT – Involved in public awareness, e.g. annual Zwelethemba Nonkululeko Big Walk PT – Awendrus Old Age Home where carers are taught to understand disabling pathologies HN – Worcester CDC, Nuwerus, Worcester Hospital regarding sensitisation of diabetic pathology and management SLHT and HN give talks on Valley FM around disease and disability awareness ALL AHS – Visit APD, NID and Institute for the Blind for their own sensitisation to issues around disability	Training regarding using the ICF – Western Cape DoH Training provided, but not accredited as provided by students Explore opportunities for collaborative tailor-made training initiatives in communities All inter-professional trainings are collaborative and based on needs analysis performed in collaboration with the community (Avian Park, Empilisweni, Hospice, ASD, APD and CDC)	There is definitely scope for more public education and training around disability in terms of awareness and sensitisation. This can be done by talks, small group discussions, role modelling disabled community members, video material at schools and for students at the RCS
12. (b) Establish and maintain a data base to be a source of information and an instrument of empowerment, e.g. in negotiations for services and lobbying for rights	No response	No response	No database existing for information
13. Enter into memorandums of understanding with communities, organisations of persons with disabilities and service providers in the interest of people with disabilities in alignment with the United Nations Convention on the Rights of Persons with Disabilities (UNCRPD and the White Paper of December 2015 on the same)	PT Students have knowledge of the UNCRPD prior to start of rotation	Ukwanda Centre for Rural Health have a Memorandum of Understanding (MOU) with Boland Hospice with regard to making use of the community care workers to help train students on the platform	Issues of consent for treatment and admission in hospitals are inconsistent at each site and for each professional body Better understanding of the UNCRPD at the RCS is important
14. Co-hosting of conferences on an annual basis	No response	Annual Rural Research Days with Anova Health Institute regarding health and wellness issues in the community and beyond has taken place annually from 2013 The DoH Cape Winelands District Health organised a rehabilitation conference hosted by the RCS which took place in November 2014	Opportunities for hosting collaborative community partnership functions where students and their community partners could present on projects that were initiated in areas around the RCS
15. (a) Sensitising and engaging (e.g. using disabled coaches, advocates, etc.) church communities and ministers/pastors to make it possible for people with disabilities to fully participate in all aspects and every level of church life (e.g. a ramp to the pulpit)	No response	No response	None – Need help from municipality and DSD Opportunity for collaboration via MSAT Invite the RHAP to present their VOICE patient advocacy workshop at the RCS for students, staff and community
15. (b) Establish a forum of people with disabilities to share information and to speak collectively on needs and human right issues	No involvement	No involvement at this stage – Except on the day this was presented	Needs to be frequent and INCLUSIVE to increase community awareness and potentially increase the SUSTAINABILITY of student run support groups Avian Park is an appropriate environment to start up forum for people with disabilities, a support group
16. Continue to involve international experts in the conference	No involvement at this stage	No involvement	No gap identified
17. More involvement of people with disabilities in future conferences as well as action plans arising from these conferences	No involvement	Persons with disabilities are specifically invited to submit an abstract, to present a workshop or presentation on building resilience in people with disabilities at the National Rural Health Conference, which was held in Worcester in 2014	
18. Look at the possibilities of establishing special interest groups for different fields and disabilities	No involvement	No involvement	No involvement at this stage Opportunities – Journal club held at the RCS open for all community. Use the current events such as community partnership function and rural research days to drive this process with the help of MSAT
19. Joint efforts by SU and Free State University training professional interpreters to assist students with disabilities to gain access to training in fields they prefer	No involvement	No involvement	Not initiated yet
20. Assist and partner with organisations working in the field of disabilities to achieve goals of empowerment	Sites that cater for persons with disabilities are involved with our students practical training which has led to reciprocal empowerment	Partnerships have been developed with sites that cater for persons with disabilities to empower both students and the site	Reciprocal capacity building joint workshops
21. Improving existing assistive devices and self-training toolkits (e.g. speech reading, lip speaking, electricity meters, etc.) for students with a diversity of hearing loss	Do not have RCS students who require these services as yet; however, there is a university mandate to cater for students with hearing loss With regard to our students’ awareness only the SLH have formal training in working with persons with hearing loss HN works at NID and receives a preparation session from the NID on working with persons with hearing loss	No involvement yet	No involvement yet The CRS has recently initiated a baseline research study looking at inclusion of assistive technology in the curriculum of rehabilitation sciences

AHS, Allied Health Sciences; APD, Association for Persons with Disabilities; ASD, Association for Persons with Sensory Disabilities; CDC, Community Day Clinic; CRS, Centre for Rehabilitation Studies; DoE, Department of Education; DSD, Department of Social Development; ECD, early childhood development; ICF, International Classification of Functioning, Disability and Health; HN, Human Nutrition; MOU, memorandum of understanding; MSAT, Multi Sectoral Action Team; NID, National Institute for the Deaf; NGO, non-governmental organisations; OT, Occupational Therapy; PT, Physiotherapy; RCS, rural clinical school; RHAP, Rural Health Advocacy Project; SLHT, Speech Language and Hearing Therapy; UNCRPD, United Nations Convention on the Rights of Persons with Disabilities; WHO, World Health Organization.

Post the responses of the coordinators, the audience which consisted of persons with disabilities, disabled people’s organisations and the academic coordinators further synthesised the needs. This is because it was clear that some of the needs were not yet being responded to through service learning. This shared dialogue led to a further synthesis of needs leading to the following themes which were highlighted as needing to be prioritised:

- *Clarify the correct terminology* to be used for persons with disabilities including disabilities related to early childhood development.
- *Include aspects of disability in the undergraduate theoretical curriculum* on all levels including research strategies to put theory into practice.
- *Research* related to mobility, access and transport.
- *Develop a database of information* on disability-related issues.
- *Share resources with provincial departments* to join hands in achieving their priorities.
- *Partner* with disabled people’s organisations and communities for capacity building and empowerment.
- *Engage with communities* on disability issues to increase sensitisation and encourage equalisation of opportunities for persons with disabilities.

Use of the nominal group technique was helpful in re-grouping and prioritising these needs with the aim to plan actions for what was not yet being addressed and strengthen what is already existing. This was followed by a discussion on steps that need to be taken as a way forward.

### The participatory response: Learning and planning for further action

A *working group* to take forward issues and suggestions arising from presentations and discussions during the 2011 conference was established, consisting of both the CRS and the RCS staff as well as the Worcester stakeholders across government and disability sectors. The aim was to mediate issues between the faculty and the community in terms of how students could engage with the community. A *disability reference group* was then established in 2015 with the following objectives:

- to validate and improve the list of needs expressed
- to play a leading role in suggesting a model for responding to these needs
- to create and maintain links or community interaction between the RCS and the community

This working group was a good response aimed at ensuring that student service learning addresses the service needs at ground level if we are to have a disability responsive curriculum. It was realised that perhaps this is a participatory cycle that should be followed by divisions to inform each curriculum review stage as needs are constantly changing. This process followed in this response could be used as a methodology for each divisional curriculum review and perhaps even work on hosting combined curriculum reviews for the four different divisions to foster both integration and inter-professional education. Such a process would allow for the emergence of curriculum directly from the community as per the social constructionist view and use this as an ideological tool as well as a vehicle for social change (Cherrington 2017).

In terms of *clarifying terminology*, the RCS used the International Classification of Functioning, Disability and Health (ICF) endorsed by the World Health Organization (2001) to facilitate holistic thinking and assessment of needs specific to persons with disabilities across the different divisions. The ICF has its own terminology that is used by the coordinators at the RCS. However, a need to clarify terminology between NGO, government, university and community stakeholders in Worcester regarding persons with disabilities was taken up by the working group. This use of the ICF not only creates uniformity across the divisions for better inter-professional education but also aligns with sectors like the health sector as well as the latest disability and rehabilitation related policies which advocate for ICF.

Of equal importance was the identification of the need for the RCS coordinators to gain insight into when and how *disability was included in the theoretical training* of the students prior to their arrival at the RCS. This would help determine if accredited workshops to keep students and staff up to date with the recent understandings and underpinnings of disability and ways of managing disability issues beyond the medical model would be necessary at the RCS, thus linking with the need for continuous capacity building through accredited workshops. Participants developed a list of topics they would like accredited workshops on and this was provided by the university through the CRS.

*Mobility, access and transport* were major issues for the sustainability of student projects due to roads being inaccessible and no transport being available for persons with disabilities. The working group supported a needs analysis of persons with disabilities in rural communities, involving access, with the support of the local municipality. The students were tasked with doing a survey in the community assessing the accessibility of facilities used by persons with disabilities and report back to the working group. These matters were then taken forward to municipal and district offices by a representative of the working group. Although the RCS campus grounds were physically accessible, no Braille, Sign Language or loop systems had been put in place. The coordinators then arranged for regular Sign Language sessions at the RCS for students to attend and a DVD on learning Sign Language was made available at the RCS library. Collaboration with the National Institute for the Deaf to involve Sign Language interpreters for conferences held at the RCS was initiated. Coordinators also made the effort to situate their students clinical training at sites where they could gain more experience from and insight into people with sensory disabilities, for example Institute for the Blind – Now called Innovation for the Blind.

*Physical resources* such as the RCS community-based service learning centre were shared with community organisations running a local soup kitchen, a swop shop, Hospice community-based care and later on a community garden club. The RCS campus was made available to outside organisations for free if the workshop or presentation spoke to issues relevant to student and staff and invited them to attend. The same agreement was made regarding talks, courses and conferences held at the RCS, which were open to community members to attend. The development of a journal club open to all community members was started in 2014 and run on a monthly basis. An annual RCS Community Partnership Function which is attended by representatives from the municipality, Cape Winelands Departments of Health, Education and Social Development and the local community since 2013 acts as fertile ground for interactive discussion around some of the community’s most pressing needs and topics presented on the day.

*Co-hosting of conferences* on an annual basis was also identified in 2011. The RCS hosts the Annual Rural Research Days with Anova Health Institute regarding health and wellness issues in the community since 2013. The event is open to all community members and offers full sponsorship for most attendees. The first Cape Winelands District Rehabilitation Conference co-hosted by the RCS took place in November 2014 and the annual Community Partnership Function is hosted at the RCS in collaboration with community partners on an annual basis. Persons with disabilities were specifically invited to attend and present on ‘Building resilience in people with disability’ at the National Rural Health Conference held in Worcester in 2014.

Education and training in communities regarding *awareness and sensitisation of disability* was an important part of advocacy and empowerment of persons with disabilities. The outcomes of these workshops were to introduce more people to disability and related issues as well as highlight how disability inclusion is everyone’s responsibility, given the complexity of challenges faced by persons with disabilities. All AHS students visited and worked at organisations servicing persons with disabilities to sensitise staff to issues around disability and to also learn from the lived experiences of persons with disabilities. Students were also involved in raising public awareness regarding disability through partnering with NGOs and/or Department of Health to present at disability and health awareness days. Since 2014, student and staff attended community organised events such as the 1000 days Indaba and Women’s Day health talks. A head injury support group was started and facilitated by the SLHT students at the local service learning centre and a therapy garden for wheelchair users was started and facilitated by the OT students. The VOICE project, presented by the Rural Health Advocacy Project (2014) on patient and health worker advocacy was hosted at the RCS twice (2015 and 2016), which served as another platform of bringing together academia and the community. In this project, there was more focus on the rights and responsibilities of persons with disabilities when it comes to victimisation, injustice or neglect. Additionally, practitioners requested accredited workshops to enable them to better respond to needs of persons with disabilities. Some of the topics that were covered include maternal health and disability; disability and sexuality and implications for the first thousand days of a child; healthcare providers’ guide to reporting healthcare challenges; building communities of practice; motivational interviewing; and understanding ethics in inter-professional teams. All workshops had an attendance of about 40–60 people.

Students also engaged with health care workers at old age homes and in the community with regard to understanding disabling pathologies and environments. Some of the divisions gave regular talks on the local radio station Valley FM. SLHT students have been involved in lip-reading strategies for people with hearing loss, while human nutrition students received sessions from the NID on working with persons with hearing loss, deaf or hard of hearing prior to their food services training. Consequently, SLHT students developed various Alternative and Augmentative Communication Boards for patients in the community to enable the family and other AHS professions to effectively communicate better during therapy. Additionally, the AHS students were working at four sites where ECD was the focus of intervention. All AHS made the effort to include parents in the early development educational interventions to further build on the existing skills of parents in raising their children with disabilities.

Community members with disabilities equally played a role in sensitising our students and staff through their collaboration and participation in RCS students’ training. Many of these community members co-presented with the students at the annual Community Partnership Function and are valued for the contribution they make to student learning.

*Research* by students specifically OT was orientated to understanding the needs of people with disabilities in rural areas and to inform practice. Student and staff conference presentations also spoke to the theory and practice involved in facilitating persons with various types of disabilities. The Division for Social Impact at SU took on the responsibility of developing and managing a virtual space where any community can approach the university to request research in a certain area of need.

### Ethical considerations

This study did not require ethical approval as no direct intervention or contact with human or animal subjects was needed.

## Discussion on practical implications

Less has been written about research which engages community stakeholders and the need to understand this as part of co-constructing a socially responsive curriculum. The methodology we followed has characteristics of what Mitchell et al. (2017) call audience research which is within participatory visual research methodologies. We argue that it may be important to recognise audience research in doing disability work in order to expand curriculum development processes to be more participatory and engaging. Additionally, it has the potential to enhance doing ‘nothing about us, without us’ in community participation for capacity building projects. The process followed here shows that engaging persons with disabilities and the community when planning for service learning has the potential to make students services more relevant and beneficial for both the university and the community. Bezzina (2019) concurs that engagements render services and development truly beneficial for persons with disabilities particularly in the Global South.

It is also apparent that academia cannot initiate or support the necessary systems and programmes required by a community if it works independently. A collective, collaborative process needs to be followed before student placement to ensure that students respond to the needs within the community. This approach will enable community members to participate actively in meeting their own needs and will serve to enhance the sustainability of responsive interventions. The consequential establishment of trustful relationships is a key to opening up spaces for dialogue and integration of diverse knowledge. In this study, a dialogue was opened between the university, the community of persons with disabilities and sectors involved in serving this community.

Furthermore, such collaborations can contribute to building greater alignment between higher education institutional curricula and the community needs while also training students on how to respectfully work with communities from a bottom up approach. It is to be noted that the approaches to collaborative action are complex and time-consuming (Doherty & Couper 2016; Pillay et al. 2016) due to the ongoing interactions between multiple stakeholders with different and sometimes opposing agendas. However, these dialogues and relationships have been documented as critical and central to ensuring culturally and contextually relevant training that speaks to the needs of the communities HEIs serve (Smith-Tolken & Bitzer 2017). Students are then also afforded the opportunity to engage in continuous learning that enables adaptation in response to changing needs in communities.

From this experience, we also argue that longer placements and more integrated rotations across the disciplines would bring a much more holistic interaction with the community thus enabling integrated responses to needs and models of best practice. It would also be critical for continuity of projects. Doherty and Couper (2016) attest to the fact that the integration of different sub-disciplines and longitudinal exposure of students to communities are central to achieving high standard rural placements. The strengthening of resources and other support provided for these efforts is thus critical in order for universities to demonstrate more social impact.

The value of the already existing inter-professional collaboration in the response to the needs of the community cannot be emphasised enough. The joint efforts of all the AHS disciplines inadvertently addressed some of the community identified needs. If each discipline had contributed independently according to their silo disciplines, it would have been impossible to meet these needs.

### Lessons learnt in facilitating reciprocity and responsivity

From the results, it is apparent that the AHS students, through their service learning on the RCS clinical training platform, are addressing some of the needs identified by persons with disabilities. However, many areas still need a stronger focus and commitment from the university. Stronger collaboration in the form of inter-professional education between the various AHS disciplines could enhance this commitment and facilitate a more comprehensive and coordinated response to the community needs through student service learning. Furthermore, such collaboration may lay a foundation for strengthened relationships and social impact between the community and the university.

Doing disability work requires reciprocity and responsiveness if we are to truly honour the inclusion saying ‘nothing about us, without us’. We learnt that reciprocity can only exist when both sides have a say and are actively participating in the collaboration. In this project, the reciprocity is evident in the sense that the community was able to express their needs to ensure that student training is servicing the hosting communities while the students were also provided with an opportunity for service learning that is community-based and community-led. The approach to the different activities facilitated a collaborative engaging process between stakeholders but equally the project was in its approach grounded, from the beginning, in principles of participation and inclusion. For instance, reflecting on the community’s needs and adjusting student’s programmes to speak to some of these needs facilitated a collaborative environment and process in which trust, accountability, co-listening, co-learning and communication were possible between the university and the community. This is to say that service learning should not only focus on upskilling the students but should also focus on serving the community in a manner that is beneficial to current needs and one that enables continuity. Given the human resource gaps, this is particularly significant in contexts where allied health practitioners are scarce at community level. This approach is integral to community-based rehabilitation principles of inclusion, participation of and giving voice to the community on how to work with communities through an empowering bottom up approach.

Additionally, reciprocity became evident as the students and staff were open to listening and learning from persons with disabilities and their lived experiences while they also willingly opened themselves to listen and learn from the staff and students. In this context, learning becomes reciprocal in the sense that everyone learns to listen to each other. This is how knowledge is produced conversationally and in relation to one another (Ned 2019). The voices of persons with disabilities are important not only because they speak from the position of having a lived experienced of disability but also because it is these voices that can deepen the understanding of doing relevant disability work. It is for such reasons that it is always important to recognise and capture the agency of persons with disabilities (Eide & Ingstad 2013). Given that disability experience is plural, recognition of agency further enriches the disability experiences.

Equally with capacity building, it was not only students whose needs were served through service learning in these communities, the project equally opened an opportunity for the disabled people’s organisations and practitioners to request accredited capacity building workshops as they saw the needs. Instead of going in with set topics of workshops from the university, the capacity building direction was fully led by the community stakeholders. This ensured that capacity building is relevant and beneficial to the community.

Each step of the interactive workshops was an opportunity for meaningful dialogic engagement between the university and the community such that all stakeholders got an opportunity to share their reflections and critique each other. These engagement processes certainly helped the coordinators in rethinking carefully about the service learning they facilitate with the students. They also deepened the understanding of the community, community dialogue and cultivated co-reflexivity. Often, reflexivity is emphasised only amongst researchers (Mitchell et al. 2017). This project demonstrated the significance of co-reflexivity amongst all involved. Maximising reflexivity between researchers, coordinators and persons with disabilities ensured that all stakeholders involved fully participated and that continuous dialogue was maintained (Mitchell et al. 2017). This shared engagement is integral to sustainability especially because community dialogue is never a once-off affair. As a result, the capacity building workshops as an immediate response extended beyond the funding term. The community and practitioners identify a topic of need and the CRS identifies a speaker for a workshop to happen.

### Limitations identified

The most significant limitation of the project was that we did not have a monitoring and evaluation tool to assess whether the needs have now changed or whether the various activities assisted to address the needs fully. Therefore, progress was difficult to assess. If we were to redo this project, we would need to develop a monitoring and evaluation tool which should be developed upfront in the participatory processes to be able to assess social impact. More persons with disabilities could be involved in the different activities, especially in capacity building workshops, so that they can facilitate these workshops. Students could also be trained to take more of a facilitator role to ensure that they do not do ‘for’ but do ‘with’ during interventions to enable full participation of persons with disabilities and continuity. This small study was not representative of all persons with disabilities in this community. A follow-up larger study with more participation of persons with disabilities, especially those with sensory impairments, intellectual disability and mental health conditions, is needed.
